# Blood Pressure Variability and Plasma Biomarkers of Neuronal Injury and Alzheimer’s Disease: A Clinic-Based Study of Patients with Diseases Along the Heart-Brain Axis

**DOI:** 10.3233/JAD-240119

**Published:** 2024-06-11

**Authors:** Naomi Louisa Paula Starmans, Laurens Jaap Kappelle, Majon Muller, Julie Staals, Charlotte Elisabeth Teunissen, Geert Jan Biessels, Wiesje Maria van der Flier, Frank Johannes Wolters, on behalf of the Heart-Brain Connection Consortium

**Affiliations:** a Department of Neurology and Neurosurgery, University Medical Center Utrecht, Utrecht, The Netherlands; b Department of Internal Medicine, Geriatrics Section, Amsterdam Cardiovascular Science, Amsterdam University Medical Center (Amsterdam UMC), Amsterdam, The Netherlands; c Department of Neurology, Cardiovascular Research Institute Maastricht (CARIM), Maastricht University Medical Center, Maastricht, The Netherlands; d Department of Clinical Chemistry, Neurochemistry Laboratory, Amsterdam Neuroscience, Amsterdam University Medical Center (Amsterdam UMC), Amsterdam, The Netherlands; e Department of Neurology, Alzheimer Center Amsterdam, Amsterdam Neuroscience, Amsterdam University Medical Center (Amsterdam UMC), Amsterdam, The Netherlands; f Department of Epidemiology, Amsterdam University Medical Center (Amsterdam UMC), Vrije Universiteit Amsterdam, Amsterdam, The Netherlands; g Department of Epidemiology, Erasmus University Medical Center, Rotterdam, The Netherlands; h Department of Radiology and Nuclear Medicine and Alzheimer Center, Erasmus University Medical Center, Rotterdam, The Netherlands

**Keywords:** Alzheimer’s disease, amyloid, biomarkers, blood pressure, dementia, orthostatic hypotension, tau proteins

## Abstract

Higher blood pressure variability (BPV) predisposes to cognitive decline. To investigate underlying mechanisms, we measured 24-h ambulatory BPV, nocturnal dipping and orthostatic hypotension in 518 participants with vascular cognitive impairment, carotid occlusive disease, heart failure, or reference participants. We determined cross-sectional associations between BPV indices and plasma biomarkers of neuronal injury (neurofilament light chain) and Alzheimer’s disease (phosphorylated-tau-181 and Aβ_42_/Aβ_40_). None of the BPV indices were significantly associated with any of the biomarkers. Hence, in patients with diseases along the heart-brain axis, we found no evidence for an association between BPV and selected markers of neuronal injury or Alzheimer’s disease.

## INTRODUCTION

Blood pressure is a highly dynamic physiological feature that varies from beat-to-beat, hour-to-hour and day-to-day, as well as upon postural change and from day to night. Various types of blood pressure variability (BPV) have been associated with an increased risk of dementia and may explain part of the association between hypertension and dementia [1–3]. Sudden or excessively low blood pressure could cause cerebral hypoperfusion, whereas high blood pressure may lead to changes in the vessel wall. Conversely, reverse causation could also explain observed associations between BPV and dementia, in which autonomic dysfunction associated with neurodegeneration leads to increased BPV.

Most studies about the link between BPV and dementia have focused on imaging markers of vascular brain injury, such as white matter hyperintensities (WMH), as outcome measures [4, 5]. However, BPV could also be related to concomitant Alzheimer’s disease (AD) pathology, since disturbances in cerebral hemodynamics accompanying BPV might contribute to these pathologies [6–8] and higher systolic BPV is associated with cognitive decline in patients with AD [9]. The recent development of plasma biomarkers may help to elucidate if BPV might indeed contribute to AD pathology specifically, in addition to the known neuronal injury through vascular or other pathways, which could be measured with the unspecific biomarker neurofilament light chain (NfL) [10, 11]. Yet, published studies on BPV and AD biomarkers have provided inconsistent results. Some studies noted that increased BPV is related to more phosphorylated-tau-181 (p-tau181) or amyloid-β (Aβ) [12–14], but not all [13, 15, 16].

We hypothesized that BPV contributes to neuronal injury (as measured by plasma NfL) and also, although potentially to a lesser extent, to AD pathology (reflected by plasma p-tau181, Aβ_40_, and Aβ_42_). We therefore determined the association between 24-h variability, nocturnal dipping or orthostatic hypotension (OH), and plasma biomarkers of neuronal injury and AD in a cohort of patients with diseases along the heart-brain axis and reference participants. By studying these hemodynamically vulnerable patients, we maximize the potential hemodynamic impact of BPV on the brain.

## MATERIALS AND METHODS

### Study population

This study was embedded in the prospective, observational Heart-Brain Connection Study. A detailed description of the rationale and study design has been published previously [17]. In short, 566 participants aged >50 years, including 437 patients with diseases along the heart-brain axis (i.e., vascular cognitive impairment (VCI), carotid occlusive disease or heart failure) as well as 129 reference participants without these conditions, were included from four participating centers between 2014 and 2019 (Supplementary Material). All participants were independent in activities of daily life and able to undergo neuropsychological assessment.

**Fig. 1:**
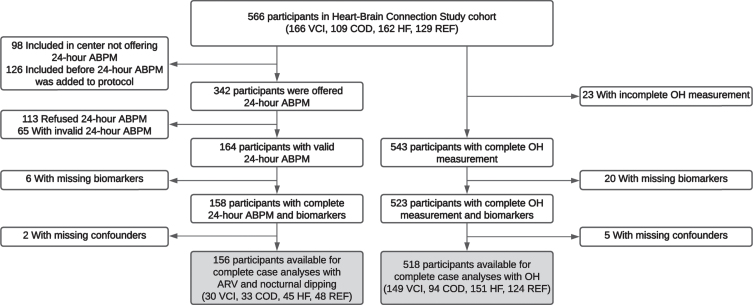
Flowchart of the inclusion of the participants. Hundred-fifty-two participants are included in both samples, 4 participants are only included in the ARV and nocturnal dipping sample and 366 participants are only included in the OH sample. ABPM, ambulatory blood pressure measurement; ARV, average real variability; COD, carotid occlusive disease; HF, heart failure; OH, orthostatic hypotension; REF, reference participants; VCI, vascular cognitive impairment.

For the present study, we included participants with the following data at baseline: 1) a valid 24-h ambulatory blood pressure measurement (ABPM) and/or a complete OH measurement and 2) a complete set of plasma biomarkers of neuronal injury and AD pathology. This resulted in a sample of 156 participants for the 24-h variability and nocturnal dipping analyses and 518 for the OH analyses (Fig. 1).

### Ethics statement

The local medical ethics committees approved the Heart-Brain Connection Study. The study was performed in accordance to the Declaration of Helsinki. All participants provided written informed consent.

### Ambulatory blood pressure measurement

From June 2015 onward, participants were offered 24-h ABPM with validated blood pressure monitors (Microlife WatchBP O3 device, Microlife Corporation, Taiwan) in three out of four participating centers (Fig. 1) [18]. The blood pressure was measured every 20 min during daytime (06.00–22.00 h) and every 60 min during nighttime (22.00–06.00 h). The 24-h ABPM was considered a valid measurement, if ≥70% of the readings were valid [19].

Mean 24-h blood pressure, 24-h variability and nocturnal dipping were then determined for both systolic (SBP) and diastolic blood pressure (DBP). We calculated 24-h variability as the 24-h, daytime (09.00–21.00 h) and nighttime (01.00–06.00 h) average real variability (ARV), with the following equation [20, 21].

$$\mathrm{ARV}=\frac{\sum \left|{\mathrm{BP}}_{k+1}-{\mathrm{BP}}_{k}\right|}{n}$$

The nocturnal dipping ratio was calculated by dividing the mean nighttime blood pressure by the mean daytime blood pressure.

### Orthostatic hypotension measurement

Participants laid down for 5 min, after which the blood pressure was measured once in the supine position. Participants then quickly got up into standing position, in which the blood pressure was measured three times consecutively, approximately at 0 s, 45 s, and 90 s after standing. The OH measurement was complete if all four blood pressure measurements were successful. OH was defined as a decrease of ≥20 mmHg systolic or ≥10 mmHg diastolic upon standing in any of the three measurements [22].

### Blood-based biomarker assessment

From non-fasting venous blood samples, we determined the plasma levels of NfL, Aβ_40_, and Aβ_42_ with the Simoa™ Neurology 4-plex E Kit and the plasma level of p-tau181 with the Simoa™ ptau181 V2 Kit on the Simoa HDX analyzer (Quanterix, Billerica, USA) in the Neurochemistry lab Amsterdam UMC (Supplementary Material). Analyses were performed in duplicates for both kits according to the manufacturer’s instructions with 1 : 4 automated on-board sample dilution. Mean intra-assay coefficients of variation were all <5%, except for a coefficient of variation of 7.4% for p-tau181. Mean inter-assay coefficients of variation were all <8%. We calculated the Aβ_42/40_ ratio by dividing the level of Aβ_42_ by the level of Aβ_40_.

### Statistical analyses

Plasma levels of NfL, p-tau181, Aβ_40_, and Aβ_42_ were log10 transformed to obtain a roughly normal data distribution. The untransformed Aβ_42/40_ ratio already had a normal data distribution. Subsequently, we standardized all biomarkers, the mean blood pressure and continuous BPV measures into z-scores.

All participants were included if they had complete data, regardless of body mass index or kidney function.

First, we determined cross-sectional associations between the various blood pressure measurements and each of the biomarkers with multiple linear regression. Both crude coefficients and coefficients adjusted for potential confounders were calculated. All analyses were adjusted for age, sex, mean 24-h SBP or DBP (depending on the determinant), use of blood pressure lowering medication and estimated glomerular filtration rate. OH analyses were additionally adjusted for the presence of diabetes mellitus (defined as a self-reported history of diabetes and/or the use of anti-diabetic medication, data on the type of diabetes was unavailable). In case of Aβ_40_ and Aβ_42_ as the outcome, analyses were also mutually adjusted for Aβ_42_ and Aβ_40_ respectively.

Next, we stratified the analyses per participant group to check for effect modification. Finally, we performed a sensitivity analysis for 24-h ARV and nocturnal dipping. The main analyses were repeated after exclusion of participants using beta-blockers as blood pressure lowering medication, since this medication is associated with the highest 24-h variability [23].

We applied false discovery rate (FDR) correction separately for each biomarker in the main analyses and stratified analyses to correct for multiple testing. A *p*-value < 0.05 after FDR correction was considered significant. Analyses were performed using R version 4.0.3.

## RESULTS

The baseline characteristics for the ARV and nocturnal dipping sample and OH sample are shown in Table 1.

**Table 1:** Baseline characteristics of the participants

Characteristic	ARV and nocturnal dipping sample *N* = 156	OH sample *N* = 518
Female	51 (32.7)	181 (34.9)
Age, y	66.1±8.3	67.9±8.6
**Participant group**
Vascular cognitive impairment	30 (19.2)	149 (28.8)
Carotid occlusive disease	33 (21.2)	94 (18.1)
Heart failure	45 (28.8)	151 (29.2)
Reference	48 (30.8)	124 (23.9)
**Blood pressure**
Mean 24-h SBP, mmHg	122.1±13.7	–
Mean 24-h DBP, mmHg	73.2±8.8	–
24-h systolic ARV, mmHg	9.8±2.5	–
24-h diastolic ARV, mmHg	7.7±1.8	–
Systolic nocturnal dipping, ratio	0.89±0.08	–
Diastolic nocturnal dipping, ratio	0.85±0.10	–
Mean resting office SBP, mmHg	142.1±22.0	141.4±20.4
Mean resting office DBP, mmHg	82.0±11.5	80.0±10.9
OH present	37 (24.3)	158 (30.5)
**Vascular risk factors and medical history**
Current smoking	27 (17.3)	87 (16.8)
LDL-cholesterol, mmol/l	2.8±1.0	2.7±1.0
Diabetes mellitus	14 (9.0)	78 (15.1)
BMI, kg/m^2^	26.7±3.7	26.9±4.2
Obesity (BMI > 30 kg/m^2^)	28 (18.1)	106 (20.5)
History of cerebrovascular or cardiovascular disease	93 (59.6)	283 (54.6)
eGFR, ml/min/1.73 m^2^	80.4±17.6	78.9±18.5
Chronic kidney disease (eGFR < 45 ml/min/1.73 m^2^)	7 (4.5)	32 (6.2)
**Medication**
Antihypertensive medication	101 (64.7)	359 (69.3)
Lipid lowering medication	91 (58.3)	311 (60.0)
**Biomarkers**
NfL, pg/ml	16.0 (12.3 – 21.9)	18.1 (13.3 – 26.1)
P-tau181, pg/ml	1.5 (1.2 – 2.0)	1.5 (1.2 – 2.2)
Aβ_42/40_, ratio	0.07 (0.06 – 0.08)	0.07 (0.06 – 0.07)
Aβ_40_, pg/ml	95.3 (84.3 – 111.0)	99.8 (87.2 – 113.4)
Aβ_42_, pg/ml	6.4 (5.6 – 7.5)	6.5 (5.6 – 7.7)

Data are presented as *N* (%) for categorical variables and mean±SD or median (interquartile range) for continuous variables. Aβ, amyloid-β; ARV, average real variability; BMI, body mass index; DBP, diastolic blood pressure; eGFR, estimated glomerular filtration rate; LDL, low-density lipoprotein; NfL, neurofilament light chain; OH, orthostatic hypotension; p-tau181, phosphorylated-tau-181; SBP, systolic blood pressure.

### Neurofilament light chain

Higher 24-h mean DBP was significantly associated with lower NfL in univariable analyses (β –0.178, 95% CI –0.330;–0.026). This association attenuated after adjustment for confounders, mostly due to age, and became non-significant (Table 2). Less nocturnal dipping, as reflected by a higher night/day-ratio, and presence of OH were associated with higher NfL in univariable analyses (for systolic dipping: β 0.197, 95% CI 0.048;0.346, for diastolic dipping: β 0.181, 95% CI 0.032;0.329, for OH: β 0.270, 95% CI 0.089;0.451), but this attenuated in multivariable models (Table 2). None of the ARV measures was significantly associated with NfL (Table 2, Supplementary Table 1).

**Table 2:** Associations between blood pressure and plasma biomarkers

	*N*	NfL	P-tau181	Ratio Aβ_42/40_	Aβ_40_	Aβ_42_
		Adjusted β (95% CI)	Adjusted β (95% CI)	Adjusted β (95% CI)	Adjusted β (95% CI)^d^	Adjusted β (95% CI)^d^
24-h mean BP, systolic^a^	156	–0.064 (–0.192 – 0.064)	–0.089 (–0.231 – 0.052)	–0.121 (–0.290 – 0.048)	0.063 (–0.075 – 0.201)	–0.086 (–0.202 – 0.030)
24-h mean BP, diastolic^a^	156	–0.074 (–0.208 – 0.059)	–0.064 (–0.212 – 0.085)	–0.085 (–0.263 – 0.093)	0.023 (–0.121 – 0.168)	–0.064 (–0.186 – 0.058)
24-h ARV, systolic^b^	156	0.010 (–0.124 – 0.144)	–0.043 (–0.190 – 0.105)	0.109 (–0.067 – 0.285)	–0.061 (–0.206 – 0.084)	0.122 (0.002 – 0.242)
24-h ARV diastolic^b^	156	–0.010 (–0.152 – 0.132)	–0.066 (–0.223 – 0.090)	–0.026 (–0.214 – 0.162)	0.062 (–0.092 – 0.216)	0.051 (–0.079 – 0.181)
Nocturnal dipping, systolic^b^	156	0.021 (–0.112 – 0.153)	0.068 (–0.078 – 0.214)	–0.044 (–0.220 – 0.131)	0.045 (–0.098 – 0.188)	–0.014 (–0.135 – 0.106)
Nocturnal dipping, diastolic^b^	156	0.026 (–0.103 – 0.155)	–0.050 (–0.193 – 0.093)	–0.049 (–0.221 – 0.122)	0.078 (–0.061 – 0.217)	–0.022 (–0.140 – 0.098)
OH present^c^	518	0.135 (–0.017 – 0.287)	–0.077 (–0.244 – 0.090)	–0.081 (–0.269 – 0.107)	0.049 (–0.084 – 0.182)	–0.082 (–0.219 – 0.056)

All continuous BP estimates are presented per 1 standard deviation increase. None of the adjusted regression coefficients was statistically significant after false discovery rate correction. Aβ, amyloid-β; ARV, average real variability; BP, blood pressure; eGFR, estimated glomerular filtration rate; NfL, neurofilament light chain; OH, orthostatic hypotension; p-tau181, phosphorylated-tau-181. ^a^Adjusted for age, sex, use of blood pressure lowering medication and eGFR. ^b^Adjusted for age, sex, mean 24-h systolic or diastolic BP (depending on the determinant), use of blood pressure lowering medication and eGFR. ^c^Adjusted for age, sex, mean 24-h systolic or diastolic BP (depending on the determinant), use of blood pressure lowering medication, eGFR and presence of diabetes mellitus. ^d^Aβ_40_ and Aβ_42_ analyses are additionally mutually adjusted for Aβ_42_ and Aβ_40_ respectively.

Results were similar across participant groups, and unaffected by exclusion of 56 participants using beta-blockers (data not shown).

### Amyloid-β and phosphorylated-tau-181

Mean blood pressure was not associated with p-tau181, the Aβ_42/40_ ratio or the separate Aβ_40_ and Aβ_42_ levels (Table 2). Regarding BPV, a higher systolic nocturnal dipping ratio was significantly associated with increased p-tau181 in the univariable analyses (β 0.254, 95% CI 0.091;0.417), but not multivariable analyses (Table 2). None of the ARV measures nor the presence of OH were significantly related to p-tau181, the Aβ_42/40_ ratio or the Aβ_40_ or Aβ_42_ level (Table 2, Supplementary Table 1).

Associations were again similar across participant groups, except for less nocturnal dipping with lower levels of Aβ_42_ among participants with VCI (for systolic dipping ratio: β –0.189, 95% CI –0.321;–0.058, for diastolic dipping ratio: β –0.208, 95% CI –0.346;–0.070), although the group-interaction was not statistically significant (p-interaction = 0.428). When excluding participants using beta-blockers, the effect estimate for the association between 24-h diastolic ARV and Aβ_40_ became slightly larger (adjusted β 0.139, 95% CI 0.005–0.273), whereas effect estimates for nocturnal dipping and the other plasma biomarkers were unchanged (data not shown).

## DISCUSSION

In this study of patients with diseases along the heart-brain axis and reference participants, ARV, nocturnal dipping and OH were overall not associated with biomarkers of neuronal injury (NfL) or AD specific biomarkers (p-tau181 and Aβ). Only in the subgroup of patients with VCI, those with less nocturnal dipping tended to have lower plasma levels of Aβ_42_.

Given the abundance of evidence linking BPV to risk of dementia and WMH [4, 5], we had anticipated an association between BPV and axonal injury, as measured by NfL. The absence of proof for an association of ARV, nocturnal dipping and OH with NfL in the current study is no proof of absence, and might be due to lack of power in the ARV and nocturnal dipping analyses or differences in study populations compared to earlier studies. A single study has previously examined the association between OH and NfL in 70 patients with Parkinson’s disease and showed that patients with OH had higher levels of plasma NfL independent of potential confounders [24]. While we also found that the presence of OH was related to higher NfL in univariable analyses, this association became non-significant after adjustment for confounders, mostly due to age. These different results may be explained by reverse causation in the prior study as OH occurs relatively late in the course of Parkinson’s disease [25], at which time NfL also increases [26, 27].

Several prior studies have investigated associations between BPV and tau or Aβ, some of which found significant associations [12–14, 28–30], whereas others did not [15, 16, 31]. These discrepancies may be explained by substantial differences in study populations, the choice of BPV index, and the technique for biomarker measurements across studies, which hamper proper comparison of study results.

More advanced stages of neurodegeneration may lead to higher BPV. Hence, study of patients with mild cognitive impairment or dementia could show more profound associations of BPV with tau or Aβ than studies among cognitively healthy individuals, due to reverse causation. Indeed, a large Chinese community-based cohort of 1546 individuals found no associations with either cerebrospinal fluid (CSF) p-tau181 or Aβ [15], whereas several studies among patients with mild cognitive impairment did [12, 14, 28, 29]. In line with this reasoning, we observed an association between nocturnal dipping and Aβ_42_ only in participants with VCI. Alternatively to reverse causation, the more profound findings in cognitively impaired individuals could be related to a higher prevalence of the *APOE* *ɛ*4 allele among these groups, which modified the associations of BPV with tau burden in ADNI participants [12, 28]. This aligns with the observation that BPV may be related to cognitive changes predominantly in *APOE* *ɛ*4 carriers [32]. Effect modification by *APO*E genotype has similarly been suggested for other demographic and vascular determinants of dementia [33].

Besides, the intensity of blood pressure treatment could be another relevant factor. Among individuals at risk for cardiovascular disease, higher BPV was associated with increased plasma total tau in the standard, but not intensive, blood pressure treatment group [30]. In our cohort of patients with overt cardiovascular disease, effect modification analyses might have revealed significant associations, although all patients were treated similarly according to current guidelines and differences between treatment groups would have likely been small.

Associations of BPV with neurodegeneration markers may depend on the choice of BPV index. Visit-to-visit variability is relatively consistently linked to AD biomarkers across different patient populations [12, 28, 29, 31], whereas shorter-term BPV (i.e., day-to-day or 24-h) generally did not relate to tau or Aβ in previous studies [15, 16], including this current report. Yet, minute-to-minute variability has been associated with plasma Aβ, though not p-tau181, among 54 community-dwelling participants with a low vascular disease burden [13]. We had no measures available for BPV other than 24-h variability. Future studies would benefit from consistent definitions of BPV and comparison of multiple BPV indices, and perhaps also pulse pressure [34], within the same population, as different types of BPV may well have distinct underlying mechanisms and downstream effects [35].

A final potentially important cause of heterogeneity between studies could be the differences in the biomarkers of interest. The plasma p-tau181 we assessed, correlates better with amyloid pathology than tau pathology on PET-imaging [36] and also has weaker associations with tau pathology on neuropathological examination as compared to CSF p-tau181 [37]. Although our negative findings for p-tau181 are in line with the negative findings for Aβ, this might explain why previous studies were able to find significant associations with plasma total tau, tau on PET or tau on neuropathological examination [28–30].

A strength of our study is that we combined multiple biomarkers in a single study of participants with hemodynamic vulnerability, in which the potential impact of BPV on the brain is maximized. The study is limited by the relatively small sample with available data on ARV and nocturnal dipping. Additionally, heterogeneity of the study sample may hamper the interpretation of our findings, although observations were mostly similar across the participant groups. Participation of relatively healthy individuals in the three patient groups may have led to selection bias. Furthermore, we had no information on *APOE* *ɛ*4 carriership, which was previously suggested to modify the association between BPV and AD pathology. Finally, we measured only p-tau181 and other forms of tau, such as p-tau217, p-tau231, or total tau, were unavailable.

In conclusion, we found no evidence for an association of 24-h BPV, nocturnal dipping and OH with biomarkers of neuronal injury (NfL) or AD (p-tau181 and Aβ) overall. A possible link between nocturnal dipping and Aβ_42_ in participants with VCI specifically warrants replication in future studies.

## AUTHOR CONTRIBUTIONS

Naomi Louisa Paula Starmans (Conceptualization; Data curation; Formal analysis; Writing – original draft; Writing – review & editing); Laurens Jaap Kappelle (Conceptualization; Investigation; Supervision; Writing – review & editing); Majon Muller (Conceptualization; Investigation; Writing – review & editing); Julie Staals (Conceptualization; Investigation; Writing – review & editing); Charlotte Elisabeth Teunissen (Conceptualization; Data curation; Writing – review & editing); Geert Jan Biessels (Conceptualization; Investigation; Writing – review & editing); Wiesje Maria van der Flier (Conceptualization; Investigation; Supervision; Writing – review & editing); Frank Johannes Wolters (Conceptualization; Formal analysis; Methodology; Writing – original draft; Writing – review & editing).

## Supplementary Material

Supplementary Material

## Data Availability

The data supporting the findings of this study are available on request from the corresponding author. The data are not publicly available due to privacy or ethical restrictions.

## References

[1] De Heus RAA , Tzourio C , Lee EJL , Opozda M , Vincent AD , Anstey KJ , Hofman A , Kario K , Lattanzi S , Launer LJ , Ma Y , Mahajan R , Mooijaart SP , Nagai M , Peters R , Turnbull D , Yano Y , the VARIABLE BRAIN Consortium, Claassen JAHR , Tully PJ (2021) Association between blood pressure variability with dementia and cognitive impairment: A systematic review and meta-analysis, Hypertension 78, 1478–1489. 34538105 10.1161/HYPERTENSIONAHA.121.17797 PMC8516811

[2] Gavriilaki M , Anyfanti P , Mastrogiannis K , Gavriilaki E , Lazaridis A , Kimiskidis V , Gkaliagkousi E (2023) Association between ambulatory blood pressure monitoring patterns with cognitive function and risk of dementia: A systematic review and meta-analysis, Aging Clin Exp Res 35, 745–761. 36995461 10.1007/s40520-023-02361-7 PMC10115699

[3] Min m , Shi T , Sun C , Liang M , Zhang Y , Wu Y , Sun Y (2018) The association between orthostatic hypotension and dementia: A meta-analysis of prospective cohort studies, Int J Geriatr Psychiatry 33, 1541–1547. 30247788 10.1002/gps.4964

[4] Ma Y , Song A , Viswanathan A , Blacker D , Vernooij MW , Hofman A , Papatheodorou S (2020) Blood pressure variability and cerebral small vessel disease: A systematic review and meta-analysis of population-based cohorts, Stroke 51, 82–89. 31771460 10.1161/STROKEAHA.119.026739 PMC7050788

[5] Tully PJ , Yano Y , Launer LJ , Kario K , Nagai M , Mooijaart SP , Claassen JAHR , Lattanzi S , Vincent AD , Tzourio C , on behalf of the Variability in Blood Pressure and Brain Health Consortium (2020) Association between blood pressure variability and cerebral small-vessel disease: A systematic review and meta-analysis, J Am Heart Assoc 9, e013841. 31870233 10.1161/JAHA.119.013841 PMC6988154

[6] Gupta A , Iadecola C (2015) Impaired Aβ clearance: A potential link between atherosclerosis and Alzheimer’s disease, Front Aging Neurosci 7, 115. 26136682 10.3389/fnagi.2015.00115 PMC4468824

[7] Koike MA , Green KN , Blurton-Jones M , LaFerla FM (2010) Oligomeric hypoperfusion differentially affects tau and amyloid-β, Am J Pathol 177, 300–310. 20472896 10.2353/ajpath.2010.090750 PMC2893673

[8] Qiu L , Ng G , Tan EK , Liao P , Kandiah N , Zeng L (2016) Chronic cerebral hypoperfusion enhances Tau hyperphosphorylation and reduces autophagy in Alzheimer’s disease mice, Sci Rep 6, 23964. 27050297 10.1038/srep23964 PMC4822118

[9] Lattanzi S , Luzzi S , Provinciali L , Silvestrini M (2014) Blood pressure variability predicts cognitive decline in Alzheimer’s disease patients, Neurobiol Aging 35, 2282–2287. 24856056 10.1016/j.neurobiolaging.2014.04.023

[10] Ashton NJ , Janelidze S , Al Khleifat A , Leuzy A , Van der Ende EL , Karikari TK , Benedet AL , Pascoal TA , Lleó A , Parnetti L , Galimberti D , Bonanni L , Pilotto A , Padovani A , Lycke J , Novakova L , Axelsson M , Velayudhan L , Rabinovici GD , Miller B , Pariante C , Nikkheslat N , Resnick SM , Thambisetty M , Schöll M , Fernández-Eulate G , Gil-Bea FJ , López de Munain A , Al-Chalabi A , Rosa-Neto P , Strydom A , Svenningsson P , Stomrud E , Santillo A , Aarsland D , Van Swieten JC , Palmqvist S , Zetterberg H , Blennow K , Hye A , Hansson O (2023) A multicentre validation study of the diagnostic value of plasma neurofilament light, Nat Commun 12, 3400. 10.1038/s41467-021-23620-z PMC8185001 34099648

[11] Merten N , Pinto AA , Paulsen AJ , Chen Y , Engelman CD , Hancock LM , Johnson SC , Schubert CR (2023) Associations of midlife lifestyle and health factors with long-term changes in blood-based biomarkers of Alzheimer’s disease and neurodegeneration, J Alzheimers Dis 94, 1381–1395. 37393497 10.3233/JAD-221287 PMC10461414

[12] Sible IJ , Nation DA , on behalf of Alzheimer’s Disease Neuroimaging Initiative (2022) Visit-to-visit blood pressure variability and CSF Alzheimer disease biomarkers in cognitively unimpaired and mildly impaired older adults, Neurology 98, e2446–e2453. 35418462 10.1212/WNL.0000000000200302 PMC9231834

[13] Sible IJ , Yew B , Jang JY , Alitin JPM , Li Y , Gaubert A , Nguyen A , Dutt S , Blanken AE , Ho JK , Marshall AJ , Kapoor A , Shenasa F , Rodgers KE , Sturm VE , Head E , Martini A , Nation DA (2022) Blood pressure variability and plasma Alzheimer’s disease biomarkers in older adults, Sci Rep 12, 17197. 36229634 10.1038/s41598-022-20627-4 PMC9561652

[14] Tarumi T , Harris TS , Hill C , German Z , Riley J , Tumer M , Womack KB , Kerwin DR , Monson NL , Stowe AM , Mathews D , Cullum CM , Zhang R (2015) Amyloid burden and sleep blood pressure in amnestic mild cognitive impairment, Neurology 85, 1922–1929. 26537049 10.1212/WNL.0000000000002167 PMC4664127

[15] Hu H , Meng L , Bi YL , Zhang W , Xu W , Shen XN , Ou YN , Ma YH , Dong Q , Tan L , Yu JT (2021) Tau pathologies mediate the association of blood pressure with cognitive impairment in adults without dementia: The CABLE study, Alzheimers Dement 18, 53–64. 34031984 10.1002/alz.12377

[16] Li L , Wang W , Lian T , Guo P , He M , Zhang W , Li J , Guan H , Luo D , Zhang W , Zhang W (2022) The Influence of 24-h ambulatory blood pressure on cognitive function and neuropathological biomarker in patients with Alzheimer’s disease, Front Aging Neurosci 14, 14:909582. 10.3389/fnagi.2022.909582 PMC9257169 35813940

[17] Hooghiemstra AM , Bertens AS , Leeuwis AE , Bron EE , Bots ML , Brunner-La Rocca HP , De Craen AJM , Van der Geert RJ , Greving JP , Kappelle LJ , Niessen WJ , Van Oostenbrugge RJ , Van Osch MJP , De Roos A , Van Rossum AC , Biessels GJ , Van Buchem MA , Daemen MJAP , Van der Flier WM , on behalf of the Heart-Brain Connection Consortium (2017) The missing link in the pathophysiology of vascular cognitive impairment: Design of the Heart-Brain Study, Cerebrovasc Dis Extra 7, 140–152. 29017156 10.1159/000480738 PMC5730112

[18] Ragazzo F , Saladini F , Palatini P (2010) Validation of the Microlife WatchBP O3 device for clinic, home, and ambulatory blood pressure measurement, according to the International Protocol, Blood Press Monit 15, 59–62. 20075717 10.1097/MBP.0b013e32833531ca

[19] O’Brien E , Parati G , Stergiou G , Asmar R , Beilin L , Bilo G , Clement D , De la Sierra A , De Leeuw P , Dolan E , Fagard R , Graves J , Head GA , Imai Y , Kario K , Lurbe E , Mallion JM , Mancia G , Mengden T , Myers M , Ogedegbe G , Ohkubo T , Omboni S , Palatini P , Redon J , Ruilope LM , Shennan A , Staessen JA , vanMontfrans G , Verdecchia P , Waeber B , Wang J , Zanchetti A , Zhang Y , on behalf of the European Society of Hypertension Working Group on Blood Pressure Monitoring (2013) European Society of Hypertension Position Paper on Ambulatory Blood Pressure Monitoring, J Hypertens 31, 1731–1768. 24029863 10.1097/HJH.0b013e328363e964

[20] Parati G , Stergiou GS , Dolan E , Bilo G (2018) Blood pressure variability: Clinical relevance and application, J Clin Hypertens (Greenwhich) 20, 1133–1137. 10.1111/jch.13304 PMC8030809 30003704

[21] Yano Y (2017) Visit-to-visit blood pressure variability – what is the current challenge? Am J Hypertens 30, 112–114. 27686336 10.1093/ajh/hpw124

[22] Freeman R , Wieling W , Axelrod FB , Benditt DG , Benarroch E , Biaggioni I , Cheshire WP , Chelimsky T , Cortelli P , Gibbons CH , Goldstein DS , Hainsworth R , Hilz MJ , Jacob G , Kaufmann H , Jordan J , Lipsitz LA , Levine BD , Low PA , Mathias C , Raj SR , Robertson D , Sandroni P , Schatz I , Schondorff R , Stewart JM , Van Dijk JG (2011) Consensus statement on the definition of orthostathic hypotension, neurally mediated syncope and the postural tachycardia syndrome, Clin Auton Res 21, 69–72. 21431947 10.1007/s10286-011-0119-5

[23] Nardin C , Rattazzi M , Pauletto P (2019) Blood pressure variability and therapeutic implications in hypertension and cardiovascular diseases, High Blood Press Cardiovasc Prev 26, 353–359. 31559570 10.1007/s40292-019-00339-z PMC6825020

[24] Park DG , Kim JW , An YS , Chang J , Yoon JH (2021) Plasma neurofilament light chain level and orthostatic hypotension in early Parkinson’s disease, J Neural Transm (Vienna) 128, 1853–1861. 34568970 10.1007/s00702-021-02423-y

[25] Dommershuijsen LJ , Heshmatollah A , Mattace Raso FUS , Koudstaal PJ , Ikram MA , Ikra MK (2021) Orthostatic hypotension: A prodromal marker of Parkinson’s disease? Mov Disord 36, 164–170. 32965064 10.1002/mds.28303 PMC7891584

[26] Mollenhauer B , Dakna M , Kruse N , Galasko D , Foroud T , Zetterberg H , Schade S , Gera RG , Wang W , Gao F , Frasier M , Chahine LM , Coffey CS , Singleton AB , Simuni T , Weintraub D , Seibyl J , Toga AW , Tanner CM , Kieburtz K , Marek K , Siderowf A , Cedarbaum JM , Hutten SJ , Trenkwalder C , Graham D (2020) Validation of serum neurofilament light chain as a biomarker of Parkinson’s disease progression, Mov Disord 35, 1999–2008. 32798333 10.1002/mds.28206 PMC8017468

[27] Bäckström D , Linder J , Jakobson S , Riklund K , Zetterberg H , Blennow K , Forsgren L , Lenfeldt N (2020) NfL as a biomarker for neurodegeneration and survival in Parkinson disease, Neurology 95, e827–e838. 32680941 10.1212/WNL.0000000000010084 PMC7605503

[28] Sible IJ , Nation DA , for the Alzheimer’s Disease Neuroimaging Initiative (2022) Visit-to-visit blood pressure variability and longitudinal tau accumulation in older adults, Hypertension 79, 629–637. 34967222 10.1161/HYPERTENSIONAHA.121.18479 PMC8979412

[29] Ma Y , Blacker D , Viswanathan A , Van Veluw SJ , Bos D , Vernooij MW , Hyman BT , Tzourio C , Das S , Hofman A (2021) Visit-to-visit blood pressure variability, neuropathology, and cognitive decline, Neurology 96, e2812–e2823. 33903194 10.1212/WNL.0000000000012065 PMC8205457

[30] Sible IJ , Nation DA (2024) Blood pressure variability and plasma Alzheimer’s disease biomarkers in the SPRINT Trial, J Alzheimers Dis 97, 1851–1860. 38306042 10.3233/JAD-230930 PMC11957751

[31] Sible IJ , Bangen KJ , Blanken AE , Ho JK , Nation DA (2021) Antemortem visit-to-visit blood pressure variability predicts cerebrovascular lesion burden in autopsy-confirmed Alzheimer’s disease, J Alzheimers Dis 83, 65–75. 34250941 10.3233/JAD-210435 PMC8820262

[32] De Oliveira FF , Chen ES , Smith MC , Bertolucci PHF (2016) Associations of blood pressure with functional and cognitive changes in patients with Alzheimer’s disease, Dement Geriatr Cogn Disord 41, 314–323. 27398980 10.1159/000447585

[33] De Oliveira FF (2023) Looking behind the curtain: Patient stratification according to genetic or demographic factors may yield unexpected results in studies of neurodegenerative diseases. J Alzheimers Dis 94, 777–780. 37393510 10.3233/JAD-230561

[34] Nation DA , Edmonds EC , Bangen KJ , Delano-Wood L , Scanlon BK , Han SD , Edland SD , Salmon DP , Galasko DR , Bondi MW , for the Alzheimer’s Disease Neuroimaging Initiative Investigators (2015) Pulse pressure in relation to tau-mediated neurodegeneration, cerebral amyloidosis, and progression to dementia in very old adults, JAMA Neurol 72, 546–553. 25822631 10.1001/jamaneurol.2014.4477 PMC4428938

[35] Parati G , Ochoa JE , Lombardi C , Bilo G (2013) Assessment and management of blood-pressure variability, Nat Rev Cardiol 10, 143–155. 23399972 10.1038/nrcardio.2013.1

[36] Therriault J , Vermeiren M , Servaes S , Tissot C , Ashton NJ , Benedet AL , Karikari TK , Lantero-Rodriguez J , Brum WS , Lussier FZ , Bezgin G , Stevenson J , Rahmouni N , Kunach P , Wang YT , Fernandez-Arias J , Socualaya KQ , Macedo AC , Ferrari-Souza JP , Ferreira PCL , Bellaver B , Leffa DT , Zimmer ER , Vitali P , Soucy JP , Triana-Baltzer G , Kolb HC , Pascoal TA , Saha-Chaudhuri P , Gauthier S , Zetterberg H , Blennow K , Rosa-Neto P (2023) Association of phosphorylated tau biomarkers with amyloid positron emission tomography vs tau positron emission tomography, JAMA Neurol 80, 188–199. 36508198 10.1001/jamaneurol.2022.4485 PMC9856704

[37] Grothe MJ , Moscoso A , Ashton NJ , Karikari TK , Lantero-Rodriguez J , Snellman A , Zetterberg H , Blennow K , Schöll M , for the Alzheimer’s Disease Neuroimaging Initiative (2021) Associations of fully automated CSF and novel plasma biomarkers with Alzheimer disease neuropathology at autopsy, Neurology 97, e1229–e1242. 34266917 10.1212/WNL.0000000000012513 PMC8480485

