# Supplementary material for: Blood Pressure Variability and Plasma Biomarkers of Neuronal Injury and Alzheimer’s Disease: A Clinic-Based Study of Patients with Diseases Along the Heart-Brain Axis
Source: J Alzheimers Dis. 2024 Jun 11;99(4):1207–15. doi: 10.3233/JAD-240119 (PMC11191465; doi:10.3233/JAD-240119)
Supplement: Supplementary Material [file jad-99-jad240119-s001.pdf]

Supplementary Material

Blood Pressure Variability and Plasma Biomarkers of Neuronal Injury and Alzheimer's Disease: A Clinic-Based Study of Patients with Diseases Along the Heart-Brain Axis

SUPPLEMENTARY METHODS

Study population

The Heart-Brain Connection Study included participants aged >50 years who were independent in daily life and able to undergo neuropsychological assessment [1]. Individuals could not participate if there was clinical evidence of a neurodegenerative disease other than vascular cognitive impairment or Alzheimer's disease.

Four specific participant groups were included: three patient groups with diseases along the heart-brain axis that make them vulnerable to hemodynamic disturbances, namely vascular cognitive impairment (N=166), carotid occlusive disease (N=109), or heart failure (N=162), and a reference group without these conditions (N=129).

Additional inclusion criteria were applied for these different participant groups. Patients with vascular cognitive impairment had to have cognitive complaints with a Mini-Mental State Examination ≥20 and additionally moderate vascular brain injury on MRI or mild vascular brain injury with at least two vascular risk factors. Carotid occlusive disease was defined as a stenosis >80% or occlusion of the internal carotid artery visible on ultrasound, MR angiography, CT angiography or digital subtraction angiography, without a plan for carotid surgery. We chose a higher than conventional threshold for a significant carotid stenosis (>80% instead of ≥70%) to select patients likely to be hemodynamically vulnerable. Heart failure was diagnosed according to the European Cardiology Society guidelines of 2016, which required signs and symptoms typical of heart failure with objective evidence of a structural or functional abnormality of the heart at rest on echocardiography, irrespective of the left ventricular ejection fraction. Participants in the reference group without these three conditions were recruited through advertisements or were spouses of the patients.

Blood-based biomarker assessment

Plasma was collected from the non-fasting participants into EDTA tubes by venipuncture (K2 EDTA). The plasma was centrifuged within 2 h at 1800xg for 10 min at room temperature and then stored at -80°C in aliquots of 0.5 ml in polypropylene tubes (Sarstedt cryovials). Before analyses, we shortly thawed the samples at room temperature and centrifuged them at 10000xg for 10 min to prevent debris from influencing the measurements. We determined the plasma levels of NfL, $A\beta_{40}$, and $A\beta_{42}$ with the Simoa™ Neurology 4-plex E Kit and the plasma level of p-tau181 with the Simoa™ ptau181 V2 Kit on the Simoa HDX analyzer (Quanterix, Billerica, United States of America) in the Neurochemistry lab Amsterdam UMC. Analyses were performed in duplicates for both kits according to the manufacturer's instructions with 1:4 automated on-board sample dilution. Mean intra-assay coefficients of variation were all <5%, except for a coefficient of variation of 7.4% for p-tau181. Mean inter-assay coefficients of variation were all <8%. We calculated the $A\beta_{42/40}$ ratio by dividing the level of $A\beta_{42}$ by the level of $A\beta_{40}$.

REFERENCES

[1] Hooghiemstra AM, Bertens AS, Leeuwis AE, Bron EE, Bots ML, Brunner-La Rocca HP, De Craen AJM, Van der Geert RJ, Greving JP, Kappelle LJ, Niessen WJ, Van Oostenbrugge RJ, Van Osch MJP, De Roos A, Van Rossum AC, Biessels GJ, Van Buchem MA, Daemen MJAP, Van der Flier WM, on behalf of the Heart-Brain Connection Consortium (2017) The missing link in the pathophysiology of vascular cognitive impairment: design of the Heart-Brain Study. *Cerebrovasc Dis Extra* **7**, 140-152.

Supplementary Table 1. Associations between daytime and nighttime average real variability and plasma biomarkers

	**Crude β (95% CI)**	**Adjusted β (95% CI)[a]**
**NfL**		
Daytime ARV, systolic	0.132 (-0.018 – 0.282)	0.040 (-0.093 – 0.173)
Daytime ARV, diastolic	0.138 (-0.015 – 0.290)	0.099 (-0.034 – 0.231)
Nighttime ARV, systolic	-0.100 (-0.248 – 0.049)	-0.060 (-0.185 – 0.065)
Nighttime ARV, diastolic	-0.090 (-0.238 – 0.058)	-0.089 (-0.212 – 0.034)
**P-tau181**		
Daytime ARV, systolic	0.058 (-0.109 – 0.224)	-0.047 (-0.194 – 0.100)
Daytime ARV, diastolic	-0.008 (-0.178 – 0.161)	-0.065 (-0.212 – 0.082)
Nighttime ARV, systolic	-0.104 (-0.268 – 0.061)	-0.065 (-0.203 – 0.072)
Nighttime ARV, diastolic	-0.097 (-0.261 – 0.066)	-0.108 (-0.244 – 0.028)
**Ratio $A\beta_{42/40}$**		
Daytime ARV, systolic	-0.024 (-0.194 – 0.147)	0.099 (-0.076 – 0.275)
Daytime ARV, diastolic	-0.043 (-0.216 – 0.131)	0.000 (-0.177 – 0.177)
Nighttime ARV, systolic	-0.033 (-0.202 – 0.135)	-0.014 (-0.180 – 0.151)
Nighttime ARV, diastolic	-0.097 (-0.265 – 0.070)	-0.092 (-0.255 – 0.072)
**$A\beta_{40}$**		
Daytime ARV, systolic	0.142 (-0.047 – 0.331)	-0.060 (-0.204 – 0.085)
Daytime ARV, diastolic	0.192 (0.001 – 0.384)	0.048 (-0.097 – 0.193)
Nighttime ARV, systolic	0.089 (-0.099 – 0.277)	0.033 (-0.103 – 0.168)
Nighttime ARV, diastolic	0.054 (-0.133 – 0.241)	0.052 (-0.082 – 0.185)
**$A\beta_{42}$**		
Daytime ARV, systolic	0.115 (-0.039 – 0.270)	0.102 (-0.018 – 0.222)
Daytime ARV, diastolic	0.133 (-0.024 – 0.290)	0.049 (-0.073 – 0.172)
Nighttime ARV, systolic	0.073 (-0.081 – 0.227)	0.040 (-0.074 – 0.155)
Nighttime ARV, diastolic	0.012 (-0.142 – 0.165)	-0.002 (-0.116 – 0.111)

All continuous BP estimates are presented per 1 standard deviation increase. False discovery rate correction was not applied.

Aβ, amyloid-β; ARV, average real variability; BP, blood pressure; NfL, neurofilament light chain; p-tau181, phosphorylated-tau-181.

[a] Adjusted for age, sex, mean 24-h systolic or diastolic BP (depending on the determinant), use of blood pressure lowering medication and estimated glomerular filtration rate. $A\beta_{40}$ and $A\beta_{42}$ analyses are additionally mutually adjusted for $A\beta_{42}$ and $A\beta_{40}$ respectively.
